# The EU and China in the climate regime: exploring different pathways towards climate justice

**DOI:** 10.1007/s10308-022-00654-6

**Published:** 2023-01-05

**Authors:** Franziskus von Lucke

**Affiliations:** University of Tübingen, Tübingen, Germany

Besides having profound economic and security consequences, climate change is inextricably linked to questions of global (in)justice. For one, responsibilities for greenhouse gas (GHG) emissions have historically been and continue to be very unevenly distributed between the Global North and the Global South (Shue 1993; Caney 2010). At the same time, the most severe effects of climate change will hit developing countries and their populations much earlier and harder than wealthy industrialized states, and future generations will most likely suffer even worse consequences if present actors do not rapidly change course (Gardiner 2006; IPCC 2018). This raises numerous questions about responsibility and entitlement, which pose significant hindrances for the climate regime and which will continue to dominate the debates about climate governance in the foreseeable future (von Lucke et al. 2021; von Lucke 2021).

China and the European Union (EU) are among the biggest emitters of GHGs, both historically and currently. In line with its rapid economic development, China has increased its emissions drastically in the last few years and now has become the single largest emitter of GHGs, even surpassing the combined emissions of developed nations (BBC 2021). EU member states on the other hand have begun to lower their emissions compared to 1990 levels but as drivers of the industrial revolution have historically contributed much more to climate change and continue to be among the biggest current emitters (European Environment Agency 2022). At the same time, China and the EU are core players in the climate regime, and both have claimed to be willing to take on responsibility to lower their emissions and to tackle global climate injustices. Without their support, an effective and just regime remains impossible (Wurzel and Connelly 2011, Hilton and Kerr 2017, Yang 2022, Liu et al. 2019).

The EU has historically been an advocate of a supranational and legally binding regime and has thus particularly pushed the Kyoto Protocol with its centralized architecture and fixed emission reduction targets for industrialized states. From a global justice perspective (Eriksen 2016; Sjursen 2017), one can conceptualize this as strengthening “impartiality,” meaning an emphasis on universalist, cosmopolitan values, scientific expertise, and supranational institutions (von Lucke et al. 2021). The EU only began to change its strategy after the failure of the Copenhagen climate summit in 2009 where it encountered severe resistance especially from the USA and large developing countries including China (Groen et al. 2012). In order to regain its influence and to reach a meaningful agreement for a post-Kyoto world at COP-21 in Paris in 2015, the EU thus largely abandoned its emphasis on “targets and timetables” and on pushing legally binding emission reduction targets. Instead, it tried to act as a “leadiator” between developing and industrialized countries (Bäckstrand and Elgström 2013), started a diplomatic outreach campaign to forge alliances (Torney and Cross 2018), and accepted a higher degree of voluntariness concerning GHG reductions—in global justice terms a strengthening “mutual recognition” and “non-domination” (von Lucke et al. 2021).

China, on the other hand, had long played the card of the developing country that cannot be negated the “right to develop” and to catch up economically (Moellendorf 2015) and that does not bear much responsibility for historic emissions (Harvey and Doherty 2018). In recent years, however, it has begun to take on more responsibility (Minas 2022; Paltsev et al. 2012; Kopra 2018; Teng and Wang 2021). Thus, it agreed on a number of environmental protection measures, ramped up the transition towards renewable energy, and with its “Ecological Civilisation” concept put forward its own pathway towards tackling environmental destruction (Yang 2022; Minas 2022). Nevertheless, China has always made clear that it will not accept too strict, obligatory emission reduction targets or any other patronizing interventions in the foreseeable future, thus underlining its emphasis on a voluntary climate regime that respects state sovereignty and different pathways towards tackling climate change and climate (in)justice (Men 2014; Yang 2022).

While the EU’s and China’s stance towards the architecture of the climate regime and their preferences concerning global justice thus might differ, in the context of the negotiations towards the 2015 Paris Agreement and its aftermath—and further pushed by the absence of the USA from the climate regime during the Trump administration—they have also changed and laid new ground for cooperation. As a consequence, China and the EU have increasingly worked together when it comes to further developing the international regime (Scott 2009; Carrapatoso 2011; Christiansen et al. 2019), while domestically, the European Green Deal and China’s Ecological Civilisation concept have opened new avenues for more progressive climate policies.

What are the justice implications of these new climate policies and the EU’s and China’s engagement in the global climate regime? How do they compare? Where do we find fields of cooperation and contestation? The contributions to this special issue tackle these questions from various theoretical angles, picking up different policy aspects, and raising important points of criticism concerning the two actors and the existing literature.

## Exploring different angles of climate justice in China and the EU

A key theme in the debates about China’s international role is the question about the extent to which the country has changed concerning climate governance and tackling climate (in)justice. Two of the contributions of the special issue explore this matter further. Jilong Yang’s paper on “Understanding China’s changing engagement in global climate governance: a struggle for identity” (Yang 2022) approaches the question from a constructivist standpoint. It makes the case for focusing on China’s changing identity as an international actor as the key driver of its changed role in the climate regime, instead of mainly looking at external circumstances. Yang argues that between COP-15 in Copenhagen in 2009 and COP-21 in 2015 that led to the Paris Agreement, China has moved towards what he calls a “Yinling Leading Power identity.” This includes a departure from its once outright refusal to accept binding targets and a more cooperative, leading stance in the negotiations, and might pave the way for an intensified cooperation between China and the EU in the climate regime. He even goes so far as to claim that global climate governance has become one of China’s prototypical discursive frames in constructing its international identity in general. However, he also finds that China’s inadequate response to international expectations and lack of self-reflection in its climate policy—exemplified by the mounting critique by small and especially vulnerable developing countries—have called into question its climate leadership and new identity.

Iselin Stensdal’s and Gørild Heggelund’s contribution, “Changes in China’s climate justice perceptions: domestic and international consequences,” traces the transformation of China’s understanding of what climate justice might entail and particularly explores the paradoxical—and changing—role of China’s identity as a developing country (see also Qian Xia 2022). Resting on a content analysis of Chinese and international primary and secondary sources, the authors argue that since 2007 when climate change was made a national priority in China, the perception of what is considered fair and just concerning climate change has changed considerably. Whereas the notion of being a developing country that first had to deal with domestic challenges such as poverty and could not be bothered to take on responsibility concerning emission reductions was still strong in the beginning, China has increasingly begun to conceptualize itself as responsible global power and “ecological civilization.” Thus, while it still insists that developed nations have to bear the main burden concerning GHG reductions, it now accepts its own moral responsibility to tackle climate change and has begun to support less wealthy developing countries in the context of South-South cooperation schemes. Additionally, on the domestic level, the transformation of climate justice perceptions has helped to speed up the move towards low-carbon energy sources. While these developments are still shot through with ambiguities—for instance, the financing of coal power plants abroad—Stensdal and Heggelund nevertheless see opportunities for an increased cooperation between the EU and China on climate and energy issues.

In contrast to these China focused contributions, the paper by Sanja Petrović, Franziska Petri, and Katja Biedenkopf “The European Parliament’s shifting perspectives on climate justice with regard to China and India” (Petrović et al. 2022) takes a European perspective. It starts out from the European Parliament’s (EP) traditionally progressive role when it comes to climate policy and to considering the needs and rights of developing countries. Resting on a content analysis of plenary debates in the EP between 1996 and 2019, it then explores how narratives about climate justice in relation to China and India—representing the two largest and most GHG intensive developing countries—have changed over time. The paper finds that while mentions of climate justice only appear in 42% of speeches on foreign climate policy, they have increased over time and have become more diverse. For instance, instead of only focusing on “distributive justice,” other justice conceptions such as “recognitional justice” increasingly come into focus, reflecting a gradual rethinking of formerly Eurocentric perspectives in the EP, and possibly signifying a greater openness towards different pathways of climate abatement. In addition, over the years, Members of the European Parliament—especially those from the political right—become more critical of China and India and gradually cease to consider them as developing countries, citing their heightened contribution to GHGs and “competitive advantage” over Europe. In sum, the authors see an increasing relevance of the Parliament concerning questions of climate justice and expect its influence on shaping the EU’s internal and external climate agenda to remain high.

The three remaining papers of the special issue all take a comparative approach and mainly focus on the economic realm. Referring to the central importance of financial interventions to tackle questions of climate (in)justice, Stephen Minas’ contribution, “Financing climate justice in the European Union and China: common mechanisms, different perspectives” (Minas 2022), compares the frameworks to finance climate justice in the EU and China. He finds that while both actors have used similar tools for transitioning financing to climate-friendly investments, they do so based on very different perspectives on climate justice. On the one hand, the EU embraces a diverse set of justice principles, including substantive aspects—for instance, making sure that the financial burdens are spread fairly and championing the concept of “just transition”—and procedural considerations—e.g., the participation of civil society in decision-making processes. By contrast, Minas argues that China’s core justice conception, “ecological civilisation,” is less focused on “just transition” and intra-country distributive justice and in the end aims at improving the living standards of the Chinese people. These differences could to some extent help us to understand why climate justice considerations played an important role in the EU’s economic responses to COVID-19, while China did not take the opportunity to foster a “green recovery.”

The paper by Sirma Altun and Ceren Ergenc “The EU and China in the global climate regime: a dialectical collaboration-competition relationship” rests on a political economy framework and specifically explores three areas that the authors deem central for the global green transition: standardization, green taxonomy, and the renewables sector. Altun and Ergenc argue that EU-China relations in these areas can best be understood as a dialectical “collaboration-competition nexus” that entails moments of consensus as well as contention. In the area of standardization, they find that China’s state-centric system of standardization might challenge the EU’s longstanding normative leadership but at the same time see opportunities for a closer international alignment with a view on China’s attempts to integrate environmental standards into its foreign investments. Concerning green taxonomy and finance, the paper detects a closer collaboration between the EU and China, not least due to the more binding nature of global financial markets. Finally, when it comes to the renewable sector, the authors claim that while China has become a serious competitor for the EU especially in the field of development cooperation, European companies still readily cooperate with their Chinese counterparts. By comparing the two actors from a political economy perspective, the paper problematizes the idealistic view in the literature of the EU as a normative power and noble climate leader and instead highlights the importance of economic drivers in EU-China relations in the climate field.

Finally, Carlos R. S. Milani and Leonildes Nazar Chaves raise similar issues but take a step back and explore the EU’s and China’s role in the climate regime from a Brazilian perspective. Starting out from a Gramscian theoretical approach, their paper “How and why European and Chinese pro‑climate leadership may be challenged by their strategic economic interests in Brazil” (Milani and Chaves 2022) problematizes both the EU’s and Chinas’ claims for climate leadership. The paper does so by exploring the direct relationship of the countries with Brazil but also looks at how the EU and China behave on the global level. In the end, Milani and Chavez argue that both countries often fail to live up to their claimed normative objectives. Instead, the maintenance of commercial flows, the access to markets and primary products, and the strategic internationalization of their economies are of much more importance. Based on these findings, the authors urge us to look beyond the normative claims of powerful actors in the climate field and instead to consider their behavior in other policy areas. This often fundamentally contradicts these claims, worsens social inequalities as well as economic asymmetries, and eventually could call into question the legitimacy of the constitutive norms of the climate agenda itself.

Drawing on a range of theoretical approaches and looking at different policy areas, the contributions of this special issue once again underline that there is no universal understanding or singular political pathway towards climate justice. They also contextualize the at times monolithic view in the literature of the EU’s and China’s role in the climate regime and highlight the profound changes the two actors have gone through in the last decades. I hope that these insights will contribute to a deeper engagement between both sides with questions of climate justice—both in theory and practice—and that they might help to overcome some of the obstacles towards effectively tackling the climate crisis and the associated injustices.

## Data Availability

No new data was generated or analysed for this introduction.

## References

[CR1] Bäckstrand K, Elgström O (2013). The EU's role in climate change negotiations From Leader to ‘Leadiator’. J European Public Policy.

[CR2] BBC (2021) Report: China emissions exceed all developed nations combined. https://www.bbc.com/news/world-asia-57018837. Accessed 16 Dec 2022

[CR3] Caney S (2010). Climate change and the duties of the advantaged. Crit Rev Int Soc Pol Phil.

[CR4] Carrapatoso A (2011). Climate policy diffusion: interregional dialogue in China–EU relations. Global Change, Peace & Security.

[CR5] Christiansen T, Kirchner EJ, Wissenbach U (2019) The European Union and China. The European Union Series. Palgrave, London

[CR6] Eriksen EO (2016) Three conceptions of global political justice. GLOBUS Research Papers(1/2016)

[CR7] European Environment Agency (2022) Total greenhouse gas emission trends and projections in Europe. https://www.eea.europa.eu/ims/total-greenhouse-gas-emission-trends. Accessed 16 Dec 2022

[CR8] Gardiner SM (2006). A perfect moral storm: climate change, intergenerational ethics and the problem of moral corruption. Environ Values.

[CR9] Groen L, Niemann A, Oberthür S (2012). The EU as a global leader? The Copenhagen and Cancun UN climate change negotiations. J Contemp Eur Res.

[CR10] Harvey F, Doherty B (2018) China demands developed countries 'pay their debts' on climate change. The Guardian

[CR11] Hilton I, Kerr O (2017). The Paris Agreement: China’s ‘New Normal’ role in international climate negotiations. Climate Policy.

[CR12] IPCC (2018) Summary for policymakers of IPCC special report on global warming of 1.5°C Approved by Governments. http://www.ipcc.ch/news_and_events/pr_181008_P48_spm.shtml. Accessed 16 Oct 2018

[CR13] Kopra S (2018). China and great power responsibility for climate change.

[CR14] Liu L, Wu T, Wan Z (2019). The EU-China relationship in a new era of global climate governance. Asia Europe J.

[CR15] Men J (2014). Climate change and EU–China partnership: realist disguise or institutionalist blessing?. Asia Europe J.

[CR16] Milani CRS, Chaves LN (2022). How and why European and Chinese pro-climate leadership may be challenged by their strategic economic interests in Brazil. Asia Europe J.

[CR17] Minas S (2022). Financing climate justice in the European Union and China: common mechanisms, different perspectives. Asia Europe J.

[CR18] Moellendorf D (2015). Climate change justice. Philosophy. Compass.

[CR19] Paltsev S, Morris J, Cai Y, Karplus V, Jacoby H (2012). The role of China in mitigating climate change. Energy Economics.

[CR20] Petrović S, Petri F, Biedenkopf K (2022). The European Parliament’s shifting perspectives on climate justice with regard to China and India. Asia Europe J.

[CR21] Qian Xia L (2022) Between two worlds: the paradox and promise of China’s climate diplomacy. LSE Commentary. https://www.lse.ac.uk/granthaminstitute/news/between-two-worlds-the-paradox-and-promise-of-chinas-climate-diplomacy/. Accessed 16 Dec 2022

[CR22] Scott D (2009). Environmental issues as a ‘strategic’ key in EU–China relations. Asia Europe J.

[CR23] Shue H (1993). Subsistence emissions and luxury emissions. Law & Policy.

[CR24] Sjursen H (2017) Global justice and foreign policy: the case of the European Union. GLOBUS Research Papers 2/2017

[CR25] Teng F, Wang P (2021). The evolution of climate governance in China: drivers, features, and effectiveness. Environ Politics.

[CR26] Torney D, Cross MKD (2018) Environmental and climate diplomacy. Building Coalitions Through Persuasion. In: Adelle C, Biedenkopf K, Torney D (eds) European Union External Environmental Policy. Rules, Regulation and Governance Beyond Borders. Palgrave Macmillan, Cham, pp 39–58

[CR27] von Lucke F (2021). Principled pragmatism in climate policy? The EU and Changing Practices of Climate Justice. Polit Geogr.

[CR28] von Lucke F, Diez T, Aamodt S, Ahrens B (2021). The EU and global climate justice: normative power caught in normative battles.

[CR29] Wurzel R, Connelly J (eds) (2011) The European Union as a leader in international climate change politics. Routledge, London

[CR30] Yang J (2022). Understanding China's changing engagement in global climate governance: a struggle for identity. Asia Europe J.

